# Risk stratification analysis of recurrent myocardial infarction in Indian population using inflammatory, lipid, thrombotic and extracellular matrix remodeling markers

**DOI:** 10.21542/gcsp.2024.25

**Published:** 2024-08-01

**Authors:** Ritu Singh, Sana Tasnim, Sudhir Chandra, Roshnara PP, Ankita Choudhary, Rajni Dawar, Parul Goyal, Mukesh Kumar Meena, Jayashree Bhattacharjee, Sanjay Tyagi

**Affiliations:** 1 Department of Biochemistry, Lady Hardinge Medical College, Connaught Place, New Delhi, India; 2 Department of Cardiology, GB Pant hospital, Raj Ghat, New Delhi, India

## Abstract

Objective: Atherosclerosis is a chronic condition characterized by impaired lipid homeostasis and chronic inflammatory pathology in large and mid-sized arteries. Myocardial infarction is caused by coronary artery thrombosis in a ruptured or unstable atherosclerotic plaque. Despite the emphasis on known triggering factors, such as hypertension and dyslipidemia, adverse events following MI, such as recurrence and mortality, are still high. Therefore, it is imperative to assess potential determinants of plaque instability. We evaluated markers of inflammation, extracellular matrix (ECM) remodeling, thrombosis, and lipids in first-time and recurrent MI (RMI).

Methods: Two hundred patients diagnosed with MI within the first 24 h of the event were included in the study and categorized as first-time or recurrent MI. Serum levels of NF-κB, hs-CRP, TNF-α, IFN γ, IL-6, VCAM-1,MMP-9, stromelysin, TIMP-1, MCP-1, PAPP-A, vWF, D-dimer, PLA2, PON-1, Apo-B, Apo-A1, ox-LDL, and anti-oxidized LDL antibodies were analyzed by ELISA. We performed a multivariate logistic regression analysis for risk stratification.

Results: The mean age of first-time MI patients was 52.4 ± 25 years and that of recurrent MI patients was 55.9 ± 24.6 years. RMI patients showed significant (*p*¡0.05) upregulation of markers of inflammation (TNF-α), endothelial adhesion (VCAM-1), ECM remodeling (MMP-9, PAPP-A), and antioxidant PON-1 enzyme. First-time MI patients had significantly higher serum IL-6 and D-dimer levels than RMI patients. Risk categorization for RMI was determined at 0.5 cut-off utilizing proteomic indicators at 95% confidence interval.

Conclusion: Non-lipid factors provide substantial insights into plaque instability. Multiple markers of inflammation, thrombosis, extracellular matrix remodeling, and paroxonase-1 are reliable indicators of recurrent myocardial infarction.

## Background

Acute myocardial infarction (AMI) is characterized by multiple risk factors. In the past few decades, there has been widespread awareness, prevention, and management programs for risk factors such as smoking, hypertension, diabetes, and other lifestyle disorders contributing to poor cardiovascular health.

According to 2022 US heart and stroke statistics, there have been reports of improvement in factors such as cessation of smoking by 6%; control of diabetes (glycated hemoglobin: HbA1c < 7%), hypertension (blood pressure < 140/90 mmHg), and dyslipidemia (total cholesterol < 240 mg/dl) by 24.6%; and increase in physical activity by 10.7%^1–6^.

However, the global mortality rate of cardiovascular disease (CVD) increased by 18.7% in 2020^1^. Approximately 2.5% of AMI patients suffer from a subsequent recurrent myocardial infarction (RMI), with an alarming 50% mortality rate^7^. Asian Indians have a 20–50% greater rate of coronary artery disease (CAD)-related mortality than any other population. Although economic and social growth and its consequences, such as changed eating habits, decreased physical activity, and an increase in hypertension and diabetes, are associated with CAD prevalence in India, these conventional risk factors cannot account for this magnitude of elevated risk^8,9^.

The recommended course of action does not solely lie in more stringent control of these factors. It is necessary to review the complex process of atherosclerosis pathogenesis and the subsequent chain of events that culminate in plaque instability, MI, and its recurrence.

Atherosclerosis is a systemic disease that affects the coronary circuit and is driven by inflammation. It starts with endothelial dysfunction and oxidative stress, which make the arteries prone to atherosclerosis^10^. When oxidised LDL (ox-LDL) accumulates in the arterial wall, it triggers phagocytosis by scavenger receptors on monocytes that have been epigenetically altered, leading to persistent inflammation^11,12^.

The antioxidant mechanism of high-density lipoprotein (HDL)-associated enzymes paraoxonase-1 (PON-1) and phospholipase A2 (PLA2) reduces lipid peroxidation build-up^13,14^. Apo-A1 and Apo-B are pivotal constituents of HDL and LDL, respectively, in the maintenance of cellular cholesterol homeostasis^15–17^. The transcriptional nuclear factor kappa B subunit (NF-κB) is activated upon phagocytosis, changing inflammatory response genes and causing pro-inflammatory cytokine release, such as tumor necrosis factor-α (TNF-α), interferon-γ (IFN-γ), interleukin-6 (IL-6), and high-sensitivity C-reactive protein (hs-CRP)^18,19^. They increase the synthesis of cell adhesion molecules including vascular cell adhesion molecule-1 (VCAM-1), promoting leukocytes attachment to endothelial cells and further enhance response to ox-LDL accumulation sites, forming foam cells and plaques^18–21^. Susceptible plaques include a thin fibrous cap (< 65 µm), a significant lipid pool (>40% of the plaque), inflammatory cells (especially macrophages), outward remodeling, neovascularization, and intra-plaque hemorrhage^22^. The zinc-dependent family of endopeptidases known as extracellular matrix remodeling markers, such as matrix metallopeptidase-9 (MMP-9), tissue inhibitor of metalloproteinase-1(TIMP-1), monocyte chemoattractant protein-1 (MCP-1), stromelysin, and pregnancy-associated plasma protein-A (PAPP-A), further contribute to plaque instability^22–26^. Endothelial dysfunction eventually leads to plaque rupture and thrombus formation, culminating in adverse cardiovascular events such as acute coronary syndrome (ACS) and AMI. Thrombotic markers such as D-dimer and von Willebrand factor (vWF) indicate the onset and progression of plaque rupture^27–29^.

Although several factors contribute to the early onset of atherosclerotic lesions, the exact rationale underlying this abrupt shift to symptomatic disease remains unclear. The inadequacy of current interventional strategies that emphasize known risk factors for the development of CAD is indicated by the high prevalence of adverse cardiac events, such as AMI and RMI. The focus should be shifted to identifying precipitating factors, especially in high-risk individuals. As several hemostatic factors are strongly correlated, it is necessary to utilize a combination of easily quantifiable biomarkers that encompass the etiopathogenesis of myocardial ischemia as risk, diagnosis, and prognosis indicators.

Our primary objective was to comprehensively assess inflammatory, extracellular matrix remodeling, thrombotic, and lipid markers in patients with first-time AMI and RMI.

## Material and Methods

### Study population

The study was conducted in the Department of Biochemistry, Lady Hardinge Medical College & associated SSK Hospital in collaboration with the Department of Cardiology, G B Pant Hospital, New Delhi. Approval was granted by relevant institutional ethics committees.

The study participants included patients diagnosed with myocardial infarction within 24 h of the event. Bilingual informed consent was taken from all study participants.

Two hundred cases of myocardial infarction were enrolled in the study based on the inclusion criteria that they were confirmed cases of myocardial infarction by a treating cardiologist on the basis of clinical evaluation, electrocardiogram changes, cardiac biomarker analysis, echocardiography, and coronary angiography. Subjects with known cases of diabetes mellitus, hypertension, neoplasia, chronic liver disease, chronic kidney disease, and inflammatory diseases, such as arthritis, were excluded from the study. Patients were categorized into two groups: those with acute myocardial infarction for the first time (AMI) and those with recurrent myocardial infarction (RMI). Patients with multiple recurrences of myocardial infarction were also considered to have RMI. The subjects were further sub-categorized based on a family history of myocardial infarction in first-degree relatives.

### Sample collection and processing

Five milliliters of peripheral venous blood were collected from all participants within 24 h of MI after confirmation of the diagnosis. The samples were taken in plain and citrated vacutainers for serum and plasma respectively. Serum and plasma separation was performed at 2000 rpm for 10 min and were stored at minus 20 degrees (in suitable aliquots) before batch analysis as detailed below.

### Parameters analyzed

The analytes were categorized as follows:

**Table:** 

Category	Markers
Inflammatory markers	NF-*κ*B, hs-CRP, TNF-*α*, IFN-*γ*, IL-6, VCAM-1
Extracellular matrix remodeling markers	MMP-9, stromelysin, TIMP-1, MCP-1, PAPP-A
Thrombotic markers	vWF, D-dimer
Lipid markers	PLA2, PON-1, Apo-B, Apo-A1, ox-LDL, anti-oxidized LDL antibody

• Serum NF-κB was estimated by double-antibody sandwich enzyme-linked immunosorbent one-step process assay (ELISA) using Human Nuclear Factor- Kappa B (NF-κB) ELISA kit by Sincere Biotech, Beijing (Lot no. E180725624, Catalog No. E13821889).
• Serum hs-CRP was estimated using C-Reactive Protein HS ELISA kit by DRG Instruments GmbH, Germany (Lot no. RN-57934, Reference EIA-3954).
• Serum TNF-α was estimated using Human TNF-α ELISA kit by Diaclone SAS, France (Batch: 1100-107, Catalog No. 950.090.096).
• Serum IFN-γ was estimated using Human IFN-γ ELISA kit by Diaclone SAS, France (Batch: 1200-67, Catalog No. 950.000.096).
• Serum VCAM-1 was estimated using Human CD106 (VCAM) ELISA kit by Diaclone SAS, France (Batch: 0106-50, Catalog No. 850.580.096).
• Serum IL-6 was estimated using Human IL-6 ELISA kit by Diaclone SAS, France (Catalog No. 950.030.096).
• Serum MMP-9 was estimated using Human MMP9 ELISA kit by QAYEE-BIO, China (Lot no. 06/2018 [96T], Catalog No. QY-E02978).
• Serum stromelysin was estimated using Human MMP3 ELISA kit by QAYEE-BIO, China (Lot no. 11/2017 [96T], Catalog No. QY-E02983).
• Serum TIMP-1 was estimated using Human TIMP1 ELISA kit by QAYEE-BIO, China (Lot no. 09/2018 [96T], Catalog No. QY-E02977).
• Serum MCP-1 was estimated using Human MCP1 ELISA kit by Diaclone SAS, France (Batch: MCP1-08, Catalog No. 873.030.096).
• Serum PAPP-A was estimated using PAPPA ELISA kit by DRG Instruments GmbH, Germany (Lot no. 46K039-2, Reference EIA-2397).
• vWF levels in citrated plasma samples were estimated using REAADS von Willebrand Factor Antigen Test kit by Corgenix, USA (Lot no. VF-205, Reference No. 034-001).
• Plasma D-dimer levels was estimated using Human D Dimer ELISA kit by Sincere Biotech, Beijing (Catalog No. E13650581).
• Serum PON-1 was estimated using Human PON1 ELISA kit by Boster Biological Technology Co., LTD (Lot no. 68713109723, Code: EK1141).
• Serum APO A-1 was estimated by using Randox ELISA Kit (Catalog No. LP 2116).
• Serum APO-B levels was estimated by using Randox ELISA Kit (Catalog No. LP 2117).
• Serum ox-LDL was estimated using Human Oxidized- Low Density Lipoprotein ELISA kit by Sincere Biotech, Beijing Catalog No. E13651349).
• Serum anti ox-LDL antibody was estimated using Human anti Oxidized- Low Density Lipoprotein ELISA kit by Sincere Biotech, Beijing (Catalog No. E13652517).
• Serum PLA2 levels was estimated using Human Phospholipase A2 ELISA kit by Sincere Biotech, Beijing (Catalog No. WLJYLP1020).

The tests were performed strictly following the manufacturer’s instructions. The optical density (OD) was measured at respective wavelengths using TECAN Infinite f200 Pro ELISA plate reader and standard curve was plotted.

### Statistical analysis

Data was entered in Microsoft Excel and analyzed by SPSS version 21.0. Kolmogorov–Smirnov tested for data normality. Quantitative variables were compared using Mann–Whitney Test (skewed data) and independent T test (for normally distributed data). Multivariate logistic regression analysis was used to identify recurring MI risk variables. A *p*-value < 0.05 was considered as statistically significant.

## Results

### Patient characteristics

The study included 117 cases of first-time AMI with a mean age of 52.4 ± 25 years and 83 cases of RMI with a mean age of 55.9 ± 24.6 years. Female patients accounted for 28 cases, of which nine were cases of RMI.

54 cases of AMI and 46 cases of RMI had a family history of MI in their first-degree relative. The mean age of RMI patients during their first MI was 51.8 ± 23 years. 8.4% of RMI patients had experience of multiple events of MI.

Although all cases of RMI were on varying doses of aspirin, clopidogrel, beta-blockers and statins post previous MI event, 92.77% had taken aspirin 75mg, clopidogrel 75mg, and statin 20mg daily for an average of 4.12 ± 3 years since first MI.

Other medications taken by RMI patients include beta-blockers (87.9%), angiotensin-converting enzyme (ACE) inhibitors or angiotensin receptor blockers (ARBs) (86.7%), anticoagulants (61.4%) and thrombolytic agents (33.7%).

6 patients of RMI had undergone coronary artery bypass graft (CABG), 73 had percutaneous intervention (PCI) and 2 had conservative medical management.

61.53% of AMI patients presented with ST elevation MI (STEMI). 38 AMI cases and 28 RMI cases were inferior wall MI (IWMI), whereas 65 AMI cases and 46 RMI cases were anterior wall MI (AWMI). Emergency management of all the cases included loading dose of aspirin 600 mg, clopidogrel 300 mg and atorvastatin 40mg. Other drugs used for medical management include prasugrel (19.69%), isosorbide dinitrate (7.5%), enoxaparin (24.2%), reteplase (13.6%), frusemide (18.8%), tenecteplase (4.5%), and morphine (92.4%) for pain management.

A total of 96 cases were consumers of tobacco, of which 54.9% were smokers. 81.9% RMI patients were noted to be smokers as well. The subjects were matched on the basis of the type (STEMI *vs* NSTEMI) and location (AWMI/ IWMI/others) of MI and emergency medical management as mentioned above.

**Table 1:** Comparison of inflammatory markers in patients of first-time acute myocardial infarction (AMI) and recurrent myocardial infarction (RMI). hs-CRP in milligrams per liter (mg/L); IFN-*γ* in picograms per milliliters (pg/ml); interleukin-6 in pg/ml; NF- *κ*B in nanograms per milliliters (ng/ml); TNF-*α* in pg/ml and VCAM-1 in ng/ml. Tests of significance used to determine *p*-value have been mentioned.

Inflammatory biomarkers	AMI	RMI	*p*-value
hs-CRP (mg/L)			
Median (25th–75th percentile)	1.09 (0.389–3.859)	1.81 (0.643–3.406)	0.224 *(Mann Whitney-U test)*
IFN-*γ* (pg/ml)			
Mean ± SD	18.56 ± 7.89	15.28 ± 5.82	0.008 *(Independent T test)*
IL-6 (pg/ml)			
Median (25th–75th percentile)	21.95 (5.112–99.084)	7.42 (4.459–21.491)	0.061 *(Mann Whitney-U test)*
NF-*κ*B (ng/ml)			
Median (25th–75th percentile)	11.84 (4.842–15.063)	10.18 (6.596–13.747)	0.757 *(Mann Whitney-U test)*
TNF-*α* (pg/ml)			
Median (25th–75th percentile)	2.1 (1.329–2.507)	2.34 (2.087–3.039)	0.0008 *(Mann Whitney-U test)*
VCAM-1(ng/ml)			
Median (25th–75th percentile)	1084.66 (462.696–1276.339)	1189.87 (995.339–1346.446)	0.037 *(Mann Whitney-U test)*

### Inflammatory markers

In all cases of myocardial infarction, median serum levels of hs-CRP, IL-6, NF-κB, TNF-α and VCAM-1 were 1.31 mg/L, 11.88 pg/ml, 11.34 ng/ml, 2.19 pg/ml and 1157.37 ng/ml, respectively. The mean serum IFN-γ level was 17.35 ± 7.34 pg/ml (Table 1).

There were significantly higher levels of TNF-α and VCAM-1 in RMI cases compared to first-time AMI, whereas IFN- *γ* levels were significantly lower in RMI cases. Comparison of inflammatory markers between total number of cases, AMI and RMI cases on the basis of family history of MI in first degree relatives, showed no statistical significance.

### Lipid markers

**Figure 1.**
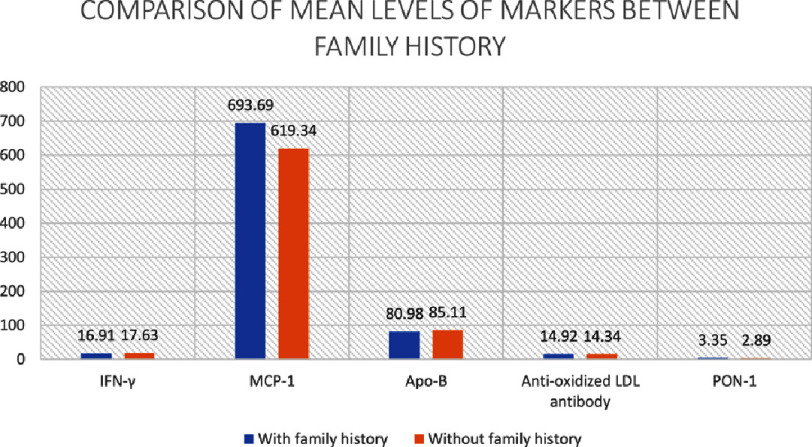
Comparison of mean levels proteomic markers. IFN-*γ* (pg/ml), MCP-1 (pg/ml), APO-B (mg/dl), anti-oxidized LDL antibody (ng/ml) and PON-1 (ng/ml) among cases of MI with and without family history of MI in first degree relative.

In all cases of myocardial infarction, median serum levels of APO-A1, ox-LDL and PLA2 were 120.36 mg/dl, 23.72 ng/ml and 4.1 ng/ml, respectively, whereas the mean serum Apo-B, anti-oxidized LDL antibody and PON-1 levels were 83.48 ± 27.98 mg/dl, 14.6 ± 1.8 ng/ml and 3.12 ± 1.07 ng/ml respectively. Among the lipid markers, only anti-oxidant enzyme PON-1 was of statistical significance. It was significantly higher in cases of RMI when compared to first-time AMI. Although not of statistical significance, Apo-A1, Apo-B, ox-LDL and PLA2 were higher and anti-oxidized LDL antibody were lower in RMI cases (Table 2). Mean PON-1 enzyme levels were significantly (*p*-value =0.061) higher in those with a family history of MI in first degree relative (3.35 ± 1.13) than in those without a family history of MI (2.89 ± 0.97) (Figure 1).

### Extracellular matrix remodeling markers

**Figure 2.**
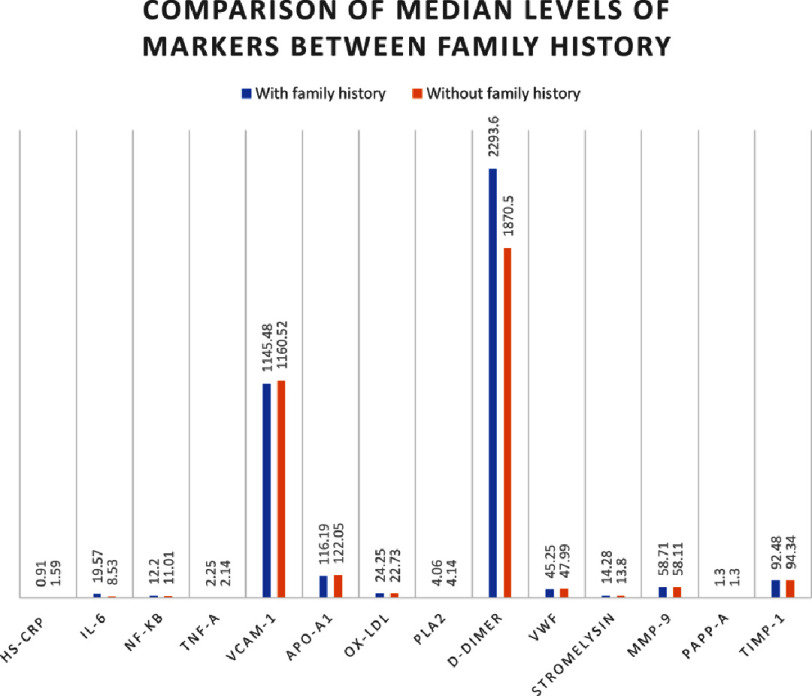
Comparison of median levels proteomic markers hs-CRP (mg/L), IL-6 (pg/ml), NF-*κ*B (ng/ml), TNF-*α* (pg/ml), VCAM-1 (ng/ml), Apo-A1 (mg/dl), ox-LDL (ng/ml), PLA2 (ng/ml),D-dimer (ng/ml), vWF (µg/ml), stromelysin (pg/ml), MMP-9 (pg/ml), PAPP-A (µg/ml) and TIMP-1 (pg/ml) among cases of MI with and without family history of MI in first degree relative.

In all cases of myocardial infarction, median serum levels of MMP-3 (stromelysin), MMP-9, PAPP-A, TIMP-1 were 14.11 pg/ml, 58.6 pg/ml, 1.3 µg/ml, 93.85 pg/ml respectively whereas the mean serum MCP-1 was 650.57 ± 625.42 pg/ml. A significantly higher level of serum MMP-9 and PAPP-A were noted in RMI cases (Table 3). No significant difference was seen between cases with respect to family history of MI in first degree relatives (Figures 1 and 2).

**Table 2:** Comparison of lipid markers in patients of first-time acute myocardial infarction (AMI) and recurrent myocardial infarction (RMI). Apo-A1 in milligrams per deciliter (mg/dl); Apo-B in mg/dl; Anti-oxidized LDL antibody in ng/ml; ox-LDL in ng/ml; PLA2 in ng/ml and PON-1 in ng/ml.

Lipid bio-markers	AMI	RMI	*p*-value
APO-A1 (mg/dL)			
Median (25th–75th percentile)	117.59 (103.462–134.995)	125.48 (106.44–135.525)	0.303 *(Mann Whitney test)*
APO-B(mg/dL)			
Mean ± SD	82.15 ± 29.14	86.4 ± 25.45	0.486 *(Independent t test)*
Anti-Oxidized LDL antibody (ng/ml)			
Mean ± SD	14.61 ± 1.69	14.59 ± 1.9	0.965 *(Independent t test)*
Ox-LDL (ng/ml)			
Median (25th–75th percentile)	22.73 (21.543–24.528)	24.81 (21.395–27.012)	0.357 *(Mann Whitney test)*
PLA2(ng/ml)			
Median (25th–75th percentile)	4.06 (4.017–4.319)	4.12 (4.013–4.28)	0.591 *(Mann Whitney test)*
PON -1 (ng/ml)			
Mean ± SD	2.78 ± 0.98	3.47 ± 1.06	0.004 *(Independent t test)*

### Thrombotic markers

The median serum levels of D-dimer and vWF in all cases of MI were 1980.8 ng/ml and 46.84 µg/ml. Cases of RMI had a significantly lower level of D-dimer compared to first-time AMI cases (Table 4). No significant difference was found in either thrombotic marker between cases with and without a history of MI in first-degree relatives (Figure 2).

Multiple regression analysis showed the possible markers of recurrence to be D-Dimer, IFN-γ, MMP-9, PAPP-A, PON-1, TNF-α and VCAM-1 (Table 5). The equation is as given:

RMI = 1/(1 +exp(.330 −  0.0004 ∗ D-dimer + 0.024 ∗ IFN-*γ* + 0.018 ∗ MMP-9 −8.245 ∗ PAPP-A + 1.214 ∗ PON-1 + 0.941 ∗ TNF-*α* + 0.003 ∗ VCAM-1)

Value < 0.5 indicates a minimal chance of recurrence in myocardial infarction, and a value >0.5 indicates a higher chance of recurrence.

**Figure 3.**
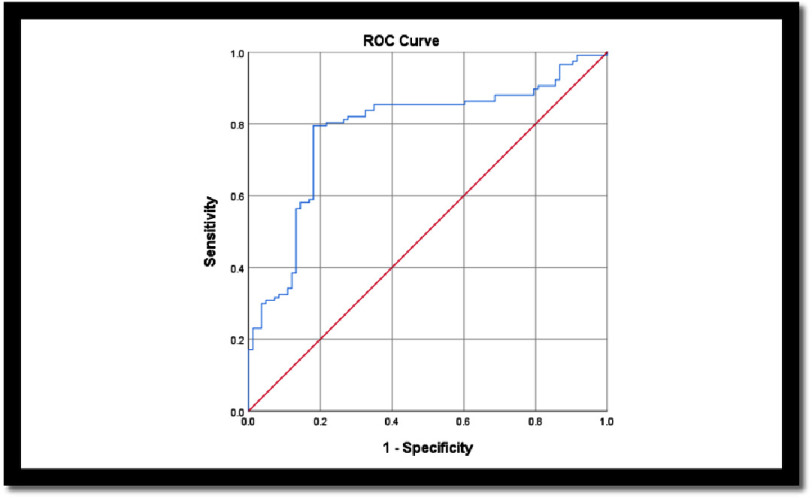
Receiver Operating Characteristic (ROC) Curve Analysis showing diagnostic performance of RMI risk algorithm in differentiating AMI and RMI.

The diagnostic performance of the RMI risk algorithm was analyzed using the receiver operating characteristic curve by area under curve (AUC) and further assessed for metrics such as sensitivity, specificity, positive predictive value, and negative predictive value (Table 6 & Figure 3). The AUC obtained was 0.784 (null hypothesis: true area = 0.5; asymptotic sig = 0.00, 95% confidence interval: lower bound = 0.718 and upper bound = 0.851).

**Table 3:** Comparison of ECM markers in patients of first-time acute myocardial infarction (AMI) and recurrent myocardial infarction (RMI). MCP-1 in pg/ml, MMP-3 in pg/ml, MMP-9, PAPP-A in ug/ml and TIMP-1 in pg/ml.

Extracellular matrix remodeling markers	AMI	RMI	p- value
MCP-1 (pg/ml)			
Mean ± SD	605.06 ± 598.27	716.98 ± 662.46	0.283 *(Independent t test)*
MMP-3 (pg/ml)			
Median (25th–75th percentile)	14.3 (12.906-20.97)	13.67 (12.917-15.125)	0.262 *(Mann WhitneyU test)*
MMP-9 (pg/ml)			
Median (25th–75th percentile)	54.56 (22.435-61.871)	60.7 (55.576-63.775)	0.0007 *(Mann Whitney U test)*
PAPP-A (ug/ml)			
Median (25th–75th percentile)	1.29 (1.275-1.317)	1.31 (1.293-1.339)	0.001 *(Mann Whitney U test)*
TIMP-1 (pg/ml)			
Median (25th–75th percentile)	97.3 (89.985-157.448)	92.34 (89.417-141.394)	0.472 *(Mann Whitney U test)*

**Table 4:** Comparison of thrombotic markers in patients of first-time acute myocardial infarction (AMI) and recurrent myocardial infarction (RMI). D-dimer in ng/ml and vWF in µg/ml.

Thrombotic markers	AMI	RMI	*p*-value
D-dimer (ng/ml)			
Median (25th–75th percentile)	2376.7 (1386.75–3349.35)	995.8 (846.975–1132.6)	< .0001 *(Mann Whitney U test)*
vWF (µg/ml)			
Median (25th–75th percentile)	47.23 (32.608–52.118)	46.77 (38.977–50.278)	0.444 *(Mann Whitney U test)*

## Discussion

According to the Global Burden of Disease study, the age-standardized death rate due to CVD in India is 272 per 100,000 individuals, notably higher than the global average of 235^30^. These figures may not be representative of the actual burden of the disease due to lack of structure in data collection and limited knowledge of the cause of death in home settings.

The conventional factors used for risk assessment of CVD in Indian medical settings are mainly LDL cholesterol levels and hyperglycemia. However, 50% of MI and strokes and 20% of significant adverse events transpire in individuals who exhibit normal LDL cholesterol levels or do not possess any identifiable risk factors.

It warrants attention that Indian population manifests CVD at a relatively young age with accelerated progression, and elevated mortality rate, surpassing that of other ethnic groups. Intriguingly, the conventional risk factors commonly do not fully elucidate the underlying reasons for this heightened susceptibility^9,31,32^. Consequently, in light of shifting atherosclerosis models, risk of adverse event may be better redefined in terms of enhanced level of plasma drivers of atherosclerosis, plaque growth and rupture activity*.* In this study, we compared 19 proteomic markers among patients with first-time AMI and RMI. The effect of long-term use of statins, aspirin and clopidogrel should be accounted for in recurrence as all patients of RMI were on these medications.

### Inflammatory markers

As a multi-functional pro-inflammatory cytokine implicated in atherosclerosis, the role of TNF-α in generating multiple chemokines like IL-6, hs-CRP and IFN-γ as acute phase response, as well as endothelial adhesion molecules such as VCAM-1 for smooth muscle proliferation, after activation through NF-κB pathway has been established by several studies^9,31,32^. An ongoing atherosclerotic process is obvious in cases of recurrence and hence, an expected increase of TNF-α and VCAM-1 is seen in cases of RMI. However, we see a decrease of IFN-γ, NF-κB, IL-6 and hs-CRP in RMI cases.

Several mechanisms have been described for non-lipid lowering effect of statins as described in Table 7.

**Table 5:** Multivariate logistic regression to find out risk factors of RMI.

	Beta coefficient	Standard error	*P*-value	Odds ratio	Odds ratio Lower bound (95%)	Odds ratio Upper bound (95%)
D-Dimer	−0.0004	0.000	0.286	1.000	0.999	1.000
IFN-*γ*	0.024	0.089	0.784	1.025	0.860	1.221
MMP-9	0.018	0.032	0.587	1.018	0.955	1.084
PAPP-A	−8.245	17.463	0.637	0.000	0.000	1.922E+11
PON-1	1.214	0.701	0.083	3.366	0.852	13.301
TNF-*α*	0.941	1.011	0.352	2.563	0.353	18.605
VCAM-1	0.003	0.002	0.254	1.003	0.998	1.007

**Table 6:** Diagnostic metrics of RMI risk algorithm.

**RMI Risk algorithm**	**Sensitivity**	**Specificity**	**PPV**	**NPV**	**AUC**
Cutoff: 0.5727 by ROC	80.17%	78.57%	83.78%	74.15%	0.784^*^

**Notes.**

* Std error = 0.034 under the non-parametric assumption.

**Table 7:** Mechanisms underlying pleotropic and immunomodulatory effect of statins.

• Inhibitory effect on NF *κ*B, suppression of inflammasome and Toll-like receptors (TLRs) and reduction in chemokines & adhesion molecules and MHC-II (by IFN-*γ*) [33,34]
• Inhibition of protein prenylation [34]
• Upregulation of endothelial nitric oxide synthase (eNOS) by attenuation of asymmetrical dimethyl arginine (ADMA), an inhibitor of the NO synthase [35,36,37]
• Activation of the PLAX2-COX pathway and production of anti-inflammatory prostacyclins [33,38]
• Downregulation of circulating endothelin-1 and subsiding endothelial dysfunction [39,40]

Lyngdoh et al. observed statins elicited a reduction in CRP levels among individuals with no discernible impact on IL-6 or TNF-*α*^41^. A 37% reduction in CRP levels was noted in subjects taking rosuvastatin by JUPITER trial^42^. CRP also exhibited a significant positive correlation with the severity of coronary stenosis and negative association with the left ventricle ejection fraction in a study by Oprescu et al. ^43^.

In a comprehensive 13-year follow-up study conducted on diabetics, it was observed that CRP exhibited a significant independent association with cardiovascular mortality^44^. Further, two meta-analyses studies concurred with the reduction of CRP, IL-6 and IFN-γ in human subjects taking statins with ezetimibe. However, no significant reduction in TNF-α was noted in these subjects^45,46^.

In cell-culture study of human umbilical cord vein endothelial cells by Bergh et al., it was shown that a potent inducing effect of TNF-α on VCAM-1 can be mitigated by simvastatin and rosuvastatin in a dose-dependant manner *via* induction of thrombomodulin and eNOS, but cannot completely counteract the effect of TNF-*α*^47^.

Our results indicate that the effect of TNF-α on VCAM-1 could not be affected by statins, although did not conclusively prove it. CRP has been a well-studied inflammatory marker in atherosclerosis as discussed above, but we found no significance of CRP in identifying recurrence.

Inhibition of cyclooxygenase (COX) and pro-inflammatory signalling cascades of NF-κB are responsible for anti-inflammatory effect of aspirin at elevated dosages. But even at lower dosages, such as prescribed maintenance dose post management of myocardial infarction, anti-inflammatory effect is seen^48,49^. The mechanisms for this effect at lower dosages have been proposed to be due to activation of thromboxane prostanoid receptor, increased activity of NO synthase and attenuation of macrophage-colony-stimulating factor, IL-6 and TNF-*α*^36,50–53^.

The effect of aspirin on circulating inflammatory markers have been contradictory. Ridker et al. in 1997 showed aspirin reduced CRP levels in MI patients, whereas in 2010, Kronish et al. showed that there was a significant negative correlation between CRP levels and aspirin adherence^54,55^.

The PLCO cancer randomised controlled trial aimed to find association of inflammatory markers with aspirin compliance in 155,000 study participants for a course of 9 years^55^. No association of CRP, IL-6 and TNF-α was found with regular aspirin use of varying dosages in this trial^56^. A trial study conducted by Block et al. showed that no significant effect of single-dose aspirin on TNF-α or IL-6^57^. But aspirin at a dosage of 160mg to 300 mg per day exhibited a notable correlation with decreased concentrations of hs-CRP, IL-6 and TNF-α by a few studies^58,59^. This shows a clear aspirin dose-dependency of inflammatory markers.

Clopidogrel is commonly used in medical management of MI owing to its P2Y_12_ platelet receptor inhibition. Anti-inflammatory function of clopidogrel has been known to be both P2Y_12_ mediated and non- P2Y_12_ mediated. Platelet-independent effect of clopidogrel has been recently shown to be because of previously thought ‘inactive’ metabolites^60^. But the impact of CYP450 genetic polymorphism on clopidogrel metabolism causing interpatient variability is significant^60,61^. Pre-clinical studies have demonstrated reduction in VCAM-1 levels and suppression of NF-κB pathway on administration of clopidogrel^62,63^. Malek et al. showed STEMI patients who were undergoing PCI had significant reduction in CRP levels on taking clopidogrel^64^. A combination of aspirin (300mg loading dose followed by 100mg per day) and clopidogrel (300mg loading dose followed by 75mg per day) for 30 days caused significant reduction in hs-CRP and TNF- α^65^.

### Lipid markers

PON-1 enzyme exhibits calcium-dependent hydrolytic activity^66^. The study conducted by Mackness et al. elucidated the functional significance of HDL-associated PON-1 in the mitigation of lipid peroxide buildup on LDL particles^67–69^. A meta-analysis study on 20 studies with 5,417 subjects showed that decreased levels of PON-1 was seen in CAD cases^70^. An inverse association between PON-1 activity and CVD-risk was established by yet another meta-analysis study^71^. Study by Singh et al. also reported a notable decrease in the concentration of serum PON-1 among patients diagnosed with myocardial MI, compared to healthy controls^72^.

In stark contrast to the anticipated diminished levels of serum PON-1 among RMI cases, the current study revealed significantly elevated levels. The observed phenomenon could potentially be explained by the mechanism of action of statins, which has been shown to modulate the expression of antioxidant enzymes. A significant correlation between increased PON-1 and arylesterase activity and statin therapy was established in a meta-analysis of 25 clinical trials^71^. An increase in enzyme concentration and activity by simvastatin was shown by treatment with simvastatin and atorvastatin^73,74^. This could be attributed to the upregulation of transcription factors, particularly sterol regulatory element binding protein-2, which leads to enhanced expression of PON-1^73^. PON-1 gene polymorphisms are known to be associated with PON-1 activity and response to statin therapy. Our results showed that patients with a family history of MI had higher PON-1 concentrations. An Indian study by Godbole et al. showed that QQ homozygosity was associated with higher PON-1 enzyme activity^75^.

### Thrombotic markers

Current research on the role of D-dimer in predicting outcomes in patients with coronary artery disease is conflicting. A previous study found that plasma D-dimer did not predict major adverse cardiovascular events (MACE) in a cohort of 3209 individuals^76^. However, the Multiethnic Study of Atherosclerosis found a significant association between elevated D-dimer levels and increased mortality risk in a cohort of over 6,000 individuals. No correlation was observed with nonfatal cardiovascular events^77^.

A retrospective study in STEMI patients found that there is a strong link between higher levels of D-dimer in the blood and an increased risk of major adverse cardiovascular events, such as heart failure, malignant arrhythmia and death^78^. The study conducted by Kikkert et al. found that having a high level of D-dimer is associated with a two times increased risk of cardiovascular events in the near-term. The study suggests that elevated levels of D-dimer upon admission are associated with an increased risk of MACE in patients with STEMI who undergo PCI^79^. Gong et al. showed that the association remains significant even after considering various factors such as age, gender, cardiovascular risk factors, and medication treatment^80^. However, our study shows that a significantly lower level of D-dimer was found in cases of RMI.

The findings of a meta-analysis indicate a correlation between the utilization of statins and a decrease in D-dimer levels, independent of both the duration of treatment and the type of statin administered. This comprehensive meta-analysis, encompassing a total of 18,052 study participants, revealed a significant decrease in D-dimer levels among individuals who underwent statin treatment compared to the control group. The overall outcome remained unaltered despite duration of treatment period and type of statin^81^.

In 156 suspected pulmonary embolism patients, Schol-Gelok et al. showed that the administration of statins was observed to be correlated with a reduction of 15% in D-dimer, as opposed to antiplatelet agents which exhibited no discernible correlation^82^. Similar results were also concluded by Alirezaei et al. in venous thromboembolism patients^83^.

### Extracellular matrix remodeling markers

The role of gelatinase B or MMP-9 in plaque instability has been extensively studied. Heightened activity of MMP-9 in unstable plaques compared to stable plaques^84–86^. MMP-9 is predominantly localized within the necrotic core and fibrous cap of atherosclerotic plaques^87–89^. Elevated levels of MMP-9 have been associated with the potential to predict plaque instability, and overexpression of MMP-9 has been linked to the induction of plaque instability^90–92^. Therefore, MMP-9 could be a promising target for mitigating the susceptibility of arterial plaques. Many studies have conclusively shown to that statins decrease MMP-9 levels by suppression of TNF-α induced macrophage infiltration^93–98^. Aspirin was found not to have any effect on MMP-9 levels^99,100^.

The administration of alteplase therapy has been observed to result in elevated concentrations of MMP-9 in individuals diagnosed with STEMI^101^. Observed increase in levels of MMP-9 indicates a potential correlation between elevated MMP activity and the ineffectiveness of thrombolytic agents as per Heo et al.^102^. The administration of anticoagulant therapy, including heparin, synthetic heparin, and direct thrombin inhibitors, has been observed to result in an elevation of MMP-9 levels in stroke patients^103,104^.

Bayes-Genis et al. established a significant association between PAPP-A and the occurrence of ACS^25^. PAPP-A functions as a protease for insulin-like growth factor-binding proteins (IGFBPs) and it was postulated that PAPP-A’s role in enhancing the local availability of insulin-like growth factor 1 (IGF-1) may potentially expedite the progression of atherosclerosis. Subsequent investigations have provided evidence linking PAPP-A to the pathogenesis of atherosclerosis^105–107^.

The CLARICOR trial sub-study 10 year follow-up investigated the relationship between PAPP-A levels and long-term mortality in individuals with stable CAD. The findings indicate that higher levels of PAPP-A are associated with increased long-term mortality in this population. However, incorporating these elevated PAPP-A levels into existing predictive models does not improve the ability to predict mortality or cardiovascular events over the long term^108^.

Comprehensive investigations regarding the impact of statins and anti-platelet medications on PAPP-A levels, especially in MI patients have yet to be conducted. The utilisation of fluvastatin as the primary therapeutic intervention for ACS in randomised, double-blind, placebo-controlled FACS-trial showed there were no statistically significant difference in PAPP-A levels observed between the group administered fluvastatin and the group administered placebo^109^. This aligns with our results as expected higher levels of PAPP-A were seen in RMI patients.

To the best of our knowledge, no study has been conducted, especially in Indian population the role of inflammatory and ECM remodeling markers in patients of RMI.

## Conclusion

Despite the effect of medications, primarily statins, antiplatelet drugs, and thrombolytic agents on numerous indicators, there was a significant difference in D-Dimer, IFN-γ, PON-1, TNF- α, and VCAM-1 levels between first-time MI and RMI patients. These had little or no influence on the ECM remodelling markers namely, MMP-9 and PAPP-A. They capture the numerous processes involved in the history of atherosclerotic plaque formation, its progression and plaque rupture and hence, enable clinicians to objectively determine an individual patient’s genuine risk for cardiovascular events and acute clinical outcomes. Because treatment cannot be discontinued in MI patients for ethical reasons, assessing the likelihood of recurrence in such patients must also take these drugs and their interactions into consideration.

## Limitations

The present investigation constituted a solitary-centre observational-analytical study, characterised by a modest sample size, thereby yielding expansive confidence intervals for certain odds ratio in the regression analysis. As such, a greater sample size would enable more conclusive findings about the utility of the markers.

The absence of a quantifiable metric for evaluating long term effect and compliance to drugs, such as statins, heightens the potential for confounding variables associated with adherence to these medications. The difference in timings of sample collection (although done within 24 h of the event) and the difference in severity of the infarction could also potentially influence the biomarker levels.

## Suggestions & future prospects

A prospective cohort study is recommended to investigate the potential effect of medications used in patients diagnosed with ACS. The study should have a long follow-up duration and aim to establish conclusive evidence on the impact of these medications on proteomic markers. Additionally comprehensive studies in more heterogenous population, such as randomized controlled trials, may be conducted to assess the effects of anti-inflammatory medications (sortilin, fontolizumab, etanercept, adalimumab) and direct MMP inhibitors (hydroxamate-based inhibitors and non-hydroxamate-based) in atherosclerosis.

## Conflict of interest

None

## Acknowledgement

This project was approved and funded by Indian Council of Medical Research (ICMR) F.No V.25011/327-HRD/2016-HR.

## References

[1] Heart Disease and Stroke Statistics—2022 Update: A Report From the American Heart Association.

[2] Smoking Cessation: A Report of the Surgeon General.

[3] Leino AD, Dorsch MP, Lester CA (2020). Changes in statin use among U.S. adults with diabetes: A population-based analysis of NHANES 2011-2018. Diabetes Care.

[4] Trends in blood pressure control among US adults with hypertension, 1999–2000 to 2017–2018 - PubMed. https://pubmed.ncbi.nlm.nih.gov/32902588/.

[5] Patel N, Bhargava A, Kalra R, Parcha V, Arora G, Muntner P, Arora P (2019). Trends in lipid, lipoproteins and statin use among U.S. Adults: Impact of 2013 cholesterol guidelines. J Am Coll Cardiol.

[6] Physical activity guidelines for Americans 2008–2018.

[7] Nair R, Johnson M, Kravitz K, Huded C, Rajeswaran J, Anabila M, Blackstone E, Menon V, Lincoff AM, Kapadia S, Khot UN (2021). Characteristics and outcomes of early recurrent myocardial infarction after acute myocardial infarction. Journal of the American Heart Association.

[8] Ralapanawa U, Sivakanesan R (2021). Epidemiology and the magnitude of coronary artery disease and acute coronary syndrome: A narrative review. J Epidemiol Glob Health.

[9] Sreeniwas Kumar A, Sinha N (2020). Cardiovascular disease in India: A 360 degree overview. Med J Armed Forces India.

[10] Thayse K, Kindt N, Laurent S, Carlier S (2020). VCAM-1 target in non-invasive imaging for the detection of atherosclerotic plaques. Biology (Basel).

[11] Wolf D, Ley K (2019). Immunity and inflammation in atherosclerosis. Circ Res.

[12] Bekkering S, Quintin J, Joosten LAB, van der Meer JWM, Netea MG, Riksen NP (2014). Oxidized low-density lipoprotein induces long-term proinflammatory cytokine production and foam cell formation via epigenetic reprogramming of monocytes. Arterioscler Thromb Vasc Biol.

[13] Mackness MI, Arrol S, Durrington PN (1991). Paraoxonase prevents accumulation of lipoperoxides in low-density lipoprotein. FEBS Lett.

[14] Dalal D, Malik AK, Dahiya K (2023). Chapter 11 - Lipoprotein-associated phospholipase A2: Antioxidant and inflammatory role. Phospholipases in Physiology and Pathology.

[15] Deng F, Li D, Lei L, Yang Q, Li Q, Wang H, Deng J, Zheng Q, Jiang W (2021). Association between apolipoprotein B/A1 ratio and coronary plaque vulnerability in patients with atherosclerotic cardiovascular disease: an intravascular optical coherence tomography study. Cardiovascular Diabetology.

[16] Sierra-Johnson J, Fisher RM, Romero-Corral A, Somers VK, Lopez-Jimenez F, Ohrvik J, Walldius G, Hellenius ML, Hamsten A (2009). Concentration of apolipoprotein B is comparable with the apolipoprotein B/apolipoprotein A-I ratio and better than routine clinical lipid measurements in predicting coronary heart disease mortality: findings from a multi-ethnic US population. Eur Heart J.

[17] Yaseen RI, El-Leboudy MH, El-Deeb HM (2021). The relation between ApoB/ApoA-1 ratio and the severity of coronary artery disease in patients with acute coronary syndrome. Egypt Heart J.

[18] Pesarini G, Amoruso A, Ferrero V, Bardelli C, Fresu LG, Perobelli L, Scappini P, De Luca G, Brunelleschi S, Vassanelli C, Ribichini F (2010). Cytokines release inhibition from activated monocytes, and reduction of in-stent neointimal growth in humans. Atherosclerosis.

[19] Liuzzo G, Santamaria M, Biasucci LM, Narducci M, Colafrancesco V, Porto A, Brugaletta S, Pinnelli M, Rizzello V, Maseri A, Crea F (2007). Persistent activation of nuclear factor kappa-B signaling pathway in patients with unstable angina and elevated levels of C-reactive protein: evidence for a direct proinflammatory effect of azide and lipopolysaccharide-free C-reactive protein on human monocytes via nuclear factor kappa-B activation. Journal of the American College of Cardiology.

[20] Moss JW, Ramji DP (2015). Interferon-*γ*: Promising therapeutic target in atherosclerosis. World J Exp Med.

[21] Bruunsgaard H, Skinhøj P, Pedersen AN, Schroll M, Pedersen BK (2000). Ageing, tumour necrosis factor-alpha (TNF-α) and atherosclerosis. Clin Exp Immunol.

[22] The role of matrix metalloproteinase-9 in atherosclerotic plaque instability. https://www.hindawi.com/journals/mi/2020/3872367/.

[23] Olejarz W, Łacheta D, Kubiak-Tomaszewska G (2020). Matrix metalloproteinases as biomarkers of atherosclerotic plaque instability. International Journal of Molecular Sciences.

[24] Loftus IM, Naylor AR, Bell PRF, Thompson MM (2002). Matrix metalloproteinases and atherosclerotic plaque instability. BJS (British Journal of Surgery).

[25] Bayes-Genis A, Conover CA, Overgaard MT, Bailey KR, Christiansen M, Holmes DR, Virmani R, Oxvig C, Schwartz RS (2001). Pregnancy-associated plasma protein A as a marker of acute coronary syndromes. N Engl J Med.

[26] Sato K, Nakano K, Katsuki S, Matoba T, Osada K, Sawamura T, Sunagawa K, Egashira K (2012). Dietary cholesterol oxidation products accelerate plaque destabilization and rupture associated with monocyte infiltration/activation via the MCP-1-CCR2 pathway in mouse brachiocephalic arteries: therapeutic effects of ezetimibe. Journal of Atherosclerosis and Thrombosis.

[27] Holm Nielsen S, Jonasson L, Kalogeropoulos K, Karsdal MA, Reese-Petersen AL, Keller Uaufdem, Genovese F, Nilsson J, Goncalves I (2020). Exploring the role of extracellular matrix proteins to develop biomarkers of plaque vulnerability and outcome. Journal of Internal Medicine.

[28] Saigo M, Hsue PY, Waters DD (2004). Role of thrombotic and fibrinolytic factors in acute coronary syndromes. Progress in Cardiovascular Diseases.

[29] Górka J, Polok K, Iwaniec T, Górka K, Włudarczyk A, Fronczek J, Devereaux PJ, Eikelboom JW, Musiał J, Szczeklik W (2017). Altered preoperative coagulation and fibrinolysis are associated with myocardial injury after non-cardiac surgery. BJA: British Journal of Anaesthesia.

[30] Prabhakaran D, Jeemon P, Roy A (2016). Cardiovascular diseases in India: current epidemiology and future directions. Circulation.

[31] Tsimikas S, Willerson JT, Ridker PM (2006). C-reactive protein and other emerging blood biomarkers to optimize risk stratification of vulnerable patients. J Am Coll Cardiol.

[32] Ruhil R (2018). India has reached on the descending limb of tobacco epidemic. Indian J Community Med.

[33] Barrientos S, Brem H, Stojadinovic O, Tomic-Canic M (2014). Clinical application of growth factors and cytokines in wound healing. Wound Repair Regen.

[34] Koushki K, Shahbaz SK, Mashayekhi K, Sadeghi M, Zayeri ZD, Taba MY, Banach M, Al-Rasadi K, Johnston TP, Sahebkar A (2021). Anti-inflammatory action of statins in cardiovascular disease: the role of inflammasome and toll-like receptor pathways. Clin Rev Allergy Immunol.

[35] Lefer DJ (2002). Statins as potent antiinflammatory drugs. Circulation.

[36] Dimitroglou Y, Aggeli C, Theofilis P, Tsioufis P, Oikonomou E, Chasikidis C, Tsioufis K, Tousoulis D (2023). Novel anti-inflammatory therapies in coronary artery disease and acute coronary syndromes. Life (Basel).

[37] Tousoulis D, Antoniades C, Vasiliadou C, Kourtellaris P, Koniari K, Marinou K, Charakida M, Ntarladimas I, Siasos G, Stefanadis C (2007). Effects of atorvastatin and vitamin C on forearm hyperaemic blood flow, asymmentrical dimethylarginine levels and the inflammatory process in patients with type 2 diabetes mellitus. Heart.

[38] Diamantis E, Kyriakos G, Quiles-Sanchez LV, Farmaki P, Troupis T (2017). The anti-inflammatory effects of statins on coronary artery disease: An updated review of the literature. Curr Cardiol Rev.

[39] Sagris M, Theofilis P, Antonopoulos AS, Oikonomou E, Paschaliori C, Galiatsatos N, Tsioufis K, Tousoulis D (2021). Inflammation in coronary microvascular dysfunction. Int J Mol Sci.

[40] Sahebkar A, Kotani K, Serban C, Ursoniu S, Mikhailidis DP, Jones SR, Ray KK, Blaha MJ, Rysz J, Toth PP, Muntner P, Lip GYH, Banach M, Lipid and Blood Pressure Meta-analysis Collaboration (LBPMC) Group (2015). Statin therapy reduces plasma endothelin-1 concentrations: A meta-analysis of 15 randomized controlled trials. Atherosclerosis.

[41] Lyngdoh T, Vollenweider P, Waeber G, Marques-Vidal P (2011). Association of statins with inflammatory cytokines: a population-based Colaus study. Atherosclerosis.

[42] Ridker PM, Danielson E, Fonseca FAH, Genest J, Gotto AM, Kastelein JJP, Koenig W, Libby P, Lorenzatti AJ, MacFadyen JG, Nordestgaard BG, Shepherd J, Willerson JT, Glynn RJ, Group JUPITERStudy (2008). Rosuvastatin to prevent vascular events in men and women with elevated C-reactive protein. N Engl J Med.

[43] Oprescu N, Micheu MM, Scafa-Udriste A, Popa-Fotea NM, Dorobantu M Inflammatory markers in acute myocardial infarction and the correlation with the severity of coronary heart disease. Ann Med.

[44] Løfblad L, Hov GG, Åsberg A, Videm V (2021). Inflammatory markers and risk of cardiovascular mortality in relation to diabetes status in the HUNT study. Sci Rep.

[45] Arabi SMostafa, Bahrami LSadat, MalekAhmadi M, Chambari M, Milkarizi N, Orekhov AN, Sahebkar A (2022). The effect of combination therapy with statins and ezetimibe on proinflammatory cytokines: A systematic review and meta-analysis of randomized controlled trials. International Immunopharmacology.

[46] Arabi SM, Chambari M, Malek-Ahmadi M, Bahrami LS, Hadi V, Rizzo M, Sahebkar A (2022). The effect of statin therapy in combination with ezetimibe on circulating C-reactive protein levels: a systematic review and meta-analysis of randomized controlled trials. Inflammopharmacol.

[47] Bergh N, Larsson P, Ulfhammer E, Jern S (2012). Effect of shear stress, statins and TNF-α on hemostatic genes in human endothelial cells. Biochemical and Biophysical Research Communications.

[48] Hohlfeld T, Schrör K (2015). Antiinflammatory effects of aspirin in ACS: relevant to its cardiocoronary actions?. Thromb Haemost.

[49] Patrono C, García Rodríguez LA, Landolfi R, Baigent C (2005). Low-dose aspirin for the prevention of atherothrombosis. N Engl J Med.

[50] Wilson SJ, Cavanagh CC, Lesher AM, Frey AJ, Russell SE, Smyth EM (2009). Activation-dependent stabilization of the human thromboxane receptor: role of reactive oxygen species. J Lipid Res.

[51] Zucker TP, Bönisch D, Muck S, Weber AA, Bretschneider E, Glusa E, Schrör K (1998). Thrombin-induced mitogenesis in coronary artery smooth muscle cells is potentiated by thromboxane A2 and involves upregulation of thromboxane receptor mRNA. Circulation.

[52] Praticò D (2008). Prostanoid and isoprostanoid pathways in atherogenesis. Atherosclerosis.

[53] Gabrielsen A, Qiu H, Bäck M, Hamberg M, Hemdahl AL, Agardh H, Folkersen L, Swedenborg J, Hedin U, Paulsson-Berne G, Haeggström JZ, Hansson GK (2010). Thromboxane synthase expression and thromboxane A2 production in the atherosclerotic lesion. J Mol Med (Berl).

[54] Kronish IM, Rieckmann N, Shimbo D, Burg M, Davidson KW (2010). Aspirin adherence, aspirin dosage, and C-reactive protein in the first three months after an acute coronary syndrome. Am J Cardiol.

[55] Ridker PM, Cushman M, Stampfer MJ, Tracy RP, Hennekens CH (1997). Inflammation, aspirin, and the risk of cardiovascular disease in apparently healthy men. N Engl J Med.

[56] Lang Kuhs KA, Hildesheim A, Trabert B, Kemp TJ, Purdue MP, Wentzensen N, Katki HA, Pinto LA, Loftfield E, Safaeian M, Chaturvedi AK, Shiels MS (2015). Association between regular aspirin use and circulating markers of inflammation: A study within the prostate, lung, colorectal and ovarian cancer screening trial. Cancer Epidemiol Biomarkers Prev.

[57] Block RC, Dier U, CalderonArtero P, Shearer GC, Kakinami L, Larson MK, Harris WS, Georas S, Mousa SA (2012). The effects of EPA+DHA and aspirin on inflammatory cytokines and angiogenesis factors. World J Cardiovasc Dis.

[58] Solheim S, Arnesen H, Eikvar L, Hurlen M, Seljeflot I (2003). Influence of aspirin on inflammatory markers in patients after acute myocardial infarction. Am J Cardiol.

[59] Gao XR, Adhikari CM, Peng LY, Guo XG, Zhai YS, He XY, Zhang LY, Lin J, Zuo ZY (2009). Efficacy of different doses of aspirin in decreasing blood levels of inflammatory markers in patients with cardiovascular metabolic syndrome. J Pharm Pharmacol.

[60] Kuszynski DS, Lauver DA (2022). Pleiotropic effects of clopidogrel. Purinergic Signal.

[61] Mega JL, Close SL, Wiviott SD, Shen L, Hockett RD, Brandt JT, Walker JR, Antman EM, Macias W, Braunwald E, Sabatine MS (2009). Cytochrome p-450 polymorphisms and response to clopidogrel. N Engl J Med.

[62] Li M, Zhang Y, Ren H, Zhang Y, Zhu X (2007). Effect of clopidogrel on the inflammatory progression of early atherosclerosis in rabbits model. Atherosclerosis.

[63] Jia Z, Huang Y, Ji X, Sun J, Fu G (2019). Ticagrelor and clopidogrel suppress NF-κB signaling pathway to alleviate LPS-induced dysfunction in vein endothelial cells. BMC Cardiovasc Disord.

[64] Malek LA, Grabowski M, Spiewak M, Filipiak KJ, Szpotanska M, Imiela T, Huczek Z, Bobilewicz D, Opolski G (2007). Relation between impaired antiplatelet response to clopidogrel and possible pleiotropic effects. J Thromb Thrombolysis.

[65] guo XUFCHENY, Y ZHANG, shang SUNYJIQ, jian LüRjuanLIR (2006). Effect of aspirin plusclopidogrel on inflammatory markers in patients with non-ST-segment elevation acutecoronary syndrome. Chinese Medical Journal.

[66] Shunmoogam N, Naidoo P, Chilton R (2018). Paraoxonase (PON)-1: a brief overview on genetics, structure, polymorphisms and clinical relevance. Vasc Health Risk Manag.

[67] Mackness MI, Arrol S, Durrington PN (1991). Paraoxonase prevents accumulation of lipoperoxides in low-density lipoprotein. FEBS Lett.

[68] Mackness MI, Arrol S, Abbott C, Durrington PN (1993). Protection of low-density lipoprotein against oxidative modification by high-density lipoprotein associated paraoxonase. Atherosclerosis.

[69] Mackness MI, Abbott C, Arrol S, Durrington PN (1993). The role of high-density lipoprotein and lipid-soluble antioxidant vitamins in inhibiting low-density lipoprotein oxidation. Biochem J.

[70] Zuin M, Trentini A, Marsillach J, D’Amuri A, Bosi C, Roncon L, Passaro A, Zuliani G, Mackness M, Cervellati C (2022). Paraoxonase-1 (PON-1) arylesterase activity levels in patients with coronary artery disease: A meta-analysis. Dis Markers.

[71] Ferretti G, Bacchetti T, Sahebkar A (2015). Effect of statin therapy on paraoxonase-1 status: A systematic review and meta-analysis of 25 clinical trials. Progress in Lipid Research.

[72] Association Of paraoxonase-1 levels and G Allele (192 A/g Polymorphism) in occurrence and recurrence of myocardial infarction, IJSR - International Journal of Scientific Research(IJSR), IJSR — World Wide Journals.

[73] Simvastatin modulates expression of the PON1 gene and increases serum paraoxonase — arteriosclerosis, thrombosis, and vascular biology. https://www.ahajournals.org/doi/10.1161/01.atv.0000096207.01487.36.

[74] Nagila A, Permpongpaiboon T, Tantrarongroj S, Porapakkham P, Chinwattana K, Deakin S,  Porntadavity S (2009). Effect of atorvastatin on paraoxonase1 (PON1) and oxidative status. Pharmacol Rep.

[75] Godbole C, Thaker S, Salagre S, Shivane V, Gogtay N, Thatte U (2023). A prospective study to assess the role of paraoxonase 1 genotype and phenotype on the lipid-lowering and antioxidant activity of statins. Indian Journal of Pharmacology.

[76] Wang TJ, Gona P, Larson MG, Tofler GH, Levy D, Newton-Cheh C, Jacques PF, Rifai N, Selhub J, Robins SJ, Benjamin EJ, D’Agostino RB, Vasan RS (2006). Multiple biomarkers for the prediction of first major cardiovascular events and death. N Engl J Med.

[77] Itakura H, Sobel BE, Boothroyd D, Leung LL, Iribarren C, Go AS, Fortmann SP, Quertermous T, Hlatky MA, Disease Atherosclerotic (2007). Vascular Function and Genetic Epidemiology Advance (ADVANCE) Study. Do plasma biomarkers of coagulation and fibrinolysis differ between patients who have experienced an acute myocardial infarction versus stable exertional angina?. Am Heart J.

[78] Zhou Q, Xue Y, Shen J, Zhou W, Wen Y, Luo S (2020). Predictive values of D-dimer for the long-term prognosis of acute ST-segment elevation infarction. Medicine (Baltimore).

[79] Kikkert WJ, Claessen BE, Stone GW, Mehran R, Witzenbichler B, Brodie BR, Wöhrle J, Witkowski A, Guagliumi G, Zmudka K, Henriques JPS, Tijssen JGP, Sanidas EA, Chantziara V, Xu K, Dangas GD (2014). D-dimer levels predict ischemic and hemorrhagic outcomes after acute myocardial infarction: a HORIZONS-AMI biomarker substudy. J Thromb Thrombolysis.

[80] Gong P, Yang SH, Li S, Luo SH, Zeng RX, Zhang Y, Guo YL, Zhu CG, Xu RX, Li JJ (2016). Plasma d-dimer as a useful marker predicts severity of atherosclerotic lesion and short-term outcome in patients with coronary artery disease. Clin Appl Thromb Hemost.

[81] Schol-Gelok S, Morelli F, Arends LR, Boersma E, Kruip MJHA, Versmissen J, van Gelder T (2019). A revised systematic review and meta-analysis on the effect of statins on D-dimer levels. Eur J Clin Invest.

[82] Schol-Gelok S, Hulle Tvander, Biedermann JS, van Gelder T, Klok FA, van der Pol LM, Versmissen J, Huisman MV, Kruip MJHA (2018). Clinical effects of antiplatelet drugs and statins on D-dimer levels. Eur J Clin Invest.

[83] Alirezaei T, Sattari H, Irilouzadian R (2022). Significant decrease in plasmad-dimer levels and mean platelet volume after a 3-month treatment with rosuvastatin in patients with venous thromboembolism. Clin Cardiol.

[84] Chen F, Eriksson P, Hansson GK, Herzfeld I, Klein M, Hansson LO, Valen G (2005). Expression of matrix metalloproteinase 9 and its regulators in the unstable coronary atherosclerotic plaque. Int J Mol Med.

[85] Park JP, Lee BK, Shim JM, Kim SH, Lee CW, Kang DH, Hong MK (2010). Relationship between multiple plasma biomarkers and vulnerable plaque determined by virtual histology intravascular ultrasound. Circ J.

[86] Liu XQ, Mao Y, Wang B, Lu XT, Bai WW, Sun YY, Liu Y, Liu HM, Zhang L, Zhao YX, Zhang Y (2014). Specific matrix metalloproteinases play different roles in intraplaque angiogenesis and plaque instability in rabbits. PLoS One.

[87] Volkov AM, Murashov IS, Polonskaya YV, Savchenko SV, Kazanskaya GM, Kliver EE, Ragino YI, CHernyavskiy AM (2018). [Changes of content of matrix metalloproteinases and their tissue expression in various types of atherosclerotic plaques]. Kardiologiia.

[88] Galis ZS, Sukhova GK, Lark MW, Libby P (1994). Increased expression of matrix metalloproteinases and matrix degrading activity in vulnerable regions of human atherosclerotic plaques. J Clin Invest.

[89] Loftus IM, Naylor AR, Goodall S, Crowther M, Jones L, Bell PR, Thompson MM (2000). Increased matrix metalloproteinase-9 activity in unstable carotid plaques. A potential role in acute plaque disruption. Stroke.

[90] Jiang XB, Wang JS, Liu DH, Yuan WS, Shi ZS (2013). Overexpression of matrix metalloproteinase-9 is correlated with carotid intraplaque hemorrhage in a swine model. J Neurointerv Surg.

[91] de Nooijer R, Verkleij CJN, von der Thüsen JH, Jukema JW, van der Wall EE, van Berkel TJC, Baker AH, a. L Biessen E (2006). Lesional overexpression of matrix metalloproteinase-9 promotes intraplaque hemorrhage in advanced lesions but not at earlier stages of atherogenesis. Arterioscler Thromb Vasc Biol.

[92] Fukuda D, Shimada K, Tanaka A, Kusuyama T, Yamashita H, Ehara S, Nakamura Y, Kawarabayashi T, Iida H, Yoshiyama M, Yoshikawa J (2006). Comparison of levels of serum matrix metalloproteinase-9 in patients with acute myocardial infarction versus unstable angina pectoris versus stable angina pectoris. Am J Cardiol.

[93] Massaro M, Zampolli A, Scoditti E, Carluccio MA, Storelli C, Distante A, De Caterina R (2010). Statins inhibit cyclooxygenase-2 and matrix metalloproteinase-9 in human endothelial cells: anti-angiogenic actions possibly contributing to plaque stability. Cardiovascular Research.

[94] Cione E, Piegari E, Gallelli G, Caroleo MC, Lamirata E, Curcio F, Colosimo F, Cannataro R, Ielapi N, Colosimo M, de Franciscis S, Gallelli L (2020). Expression of MMP-2, MMP-9, and NGAL in tissue and serum of patients with vascular aneurysms and their modulation by statin treatment: A pilot study. Biomolecules.

[95] Mohebbi N, Khoshnevisan A, Naderi S, Abdollahzade S, Salamzadeh J, Javadi M, Mojtahedzadeh M, Gholami K (2014). Effects of atorvastatin on plasma matrix metalloproteinase-9 concentration after glial tumor resection; a randomized, double blind, placebo controlled trial. Daru.

[96] Luan Z, Chase AJ, Newby AC (2003). Statins inhibit secretion of metalloproteinases-1, -2, -3, and -9 from vascular smooth muscle cells and macrophages. Arterioscler Thromb Vasc Biol.

[97] Yasuda S, Miyazaki S, Kinoshita H, Nagaya N, Kanda M, Goto Y, Nonogi H (2007). Enhanced cardiac production of matrix metalloproteinase-2 and -9 and its attenuation associated with pravastatin treatment in patients with acute myocardial infarction. Clin Sci (Lond).

[98] Turner NA, Aley PK, Hall KT, Warburton P, Galloway S, Midgley L, O’Regan DJ, Wood IC, Ball SG, Porter KE (2007). Simvastatin inhibits TNFalpha-induced invasion of human cardiac myofibroblasts via both MMP-9-dependent and -independent mechanisms. J Mol Cell Cardiol.

[99] Mehta JL, Chen J, Yu F, Li DY (2004). Aspirin inhibits ox-LDL-mediated LOX-1 expression and metalloproteinase-1 in human coronary endothelial cells. Cardiovasc Res.

[100] Kiran MS, Sameer Kumar VB, Viji RI, Sudhakaran PR (2006). Temporal relationship between MMP production and angiogenic process in HUVECs. Cell Biol Int.

[101] Tziakas DN, Chalikias GK, Parissis JT, Hatzinikolaou EI, Papadopoulos ED, Tripsiannis GA, Papadopoulou EG, Tentes IK, Karas SM, Chatseras DI (2004). Serum profiles of matrix metalloproteinases and their tissue inhibitor in patients with acute coronary syndromes. The effects of short-term atorvastatin administration. Int J Cardiol.

[102] Heo JH, Kim SH, Lee KY, Kim EH, Chu CK, Nam JM (2003). Increase in plasma matrix metalloproteinase-9 in acute stroke patients with thrombolysis failure. Stroke.

[103] Rababah M, Worthmann H, Deb M, Tryc AB, Ma YT, El Bendary OM, Hecker H, Goldbecker A, Heeren M, Brand K, Weissenborn K, Lichtinghagen R (2012). Anticoagulants affect matrix metalloproteinase 9 levels in blood samples of stroke patients and healthy controls. Clin Biochem.

[104] Castellazzi M, Tamborino C, Fainardi E, Manfrinato MC, Granieri E, Dallocchio F, Bellini T (2007). Effects of anticoagulants on the activity of gelatinases. Clin Biochem.

[105] Lund J, Qin QP, Ilva T, Pettersson K, Voipio-Pulkki LM, Porela P, Pulkki K (2003). Circulating pregnancy-associated plasma protein a predicts outcome in patients with acute coronary syndrome but no troponin I elevation. Circulation.

[106] Heider P, Pfäffle N, Pelisek J, Wildgruber M, Poppert H, Rudelius M, Eckstein HH (2010). Is serum pregnancy-associated plasma protein a really a potential marker of atherosclerotic carotid plaque stability?. European Journal of Vascular and Endovascular Surgery.

[107] Iversen KK, Dalsgaard M, Teisner AS, Schoos M, Teisner B, Nielsen H, Clemmensen P, Grande P (2009). Usefulness of pregnancy-associated plasma protein a in patients with acute coronary syndrome. American Journal of Cardiology.

[108] Nilsson E, Kastrup J, Sajadieh A, Jensen GBoje, Kjøller E, Kolmos HJ, Wuopio J, Nowak C, Larsson A, Jakobsen JC, Winkel P, Gluud C, Iversen KK, Ärnlöv J, Carlsson AC (2020). Pregnancy associated plasma protein-a as a cardiovascular risk marker in patients with stable coronary heart disease during 10 years follow-up—a CLARICOR trial sub-study. J Clin Med.

[109] Ostadal P, Alan D, Vejvoda J, Kukacka J, Macek M, Hajek P, Mates M, Kvapil M, Kettner J, Wiendl M, Aschermann O, Slaby J, Holm F, Telekes P, Horak D, Blasko P, Zemanek D, Veselka J, Cepova J (2010). Fluvastatin in the first-line therapy of acute coronary syndrome: results of the multicenter, randomized, double-blind, placebo-controlled trial (the FACS-trial). Trials.

